# Fast-Setting Permeable Alkyd/Polyester Composites: Moulding Sands

**DOI:** 10.3390/polym13244386

**Published:** 2021-12-14

**Authors:** Wojciech (Voytek) S. Gutowski, Andrzej K. Błędzki

**Affiliations:** 1 Faculty of Science, The University of Melbourne, Parkville, VIC 3010, Australia; 2 Polymeric Materials Engineering, West Pomeranian University of Technology, 70-310 Szczecin, Poland

**Keywords:** composites, moulding sands, controlled cure, alkyd/polyester, furan, core-/mould-making mixtures, heavy castings, thermal stability, low-gas emission, industrial production

## Abstract

This paper presents the outcomes of extensive research targeting the development of high-performance alkyd and polyester resins used as binders in mould- and core-making permeable composite materials designated for large-size/complex-shape, heavy alloy-steel and cast-iron castings (0.5 to 50 tonnes): steam turbine casings (e.g., 18K360 condensing turbine), naval engine blocks and heavy machinery. The technology was implemented by Zamech/ALSTON Power. The key issues discussed here are: (1) control of resin crosslinking kinetics; slow or rapid strength development, (2) shelf-life control of pre-mixed composite, (3) improved thermo-mechanical stability; (4) kinetics of gaseous by-product emission. Optimised composite formulations (resins, crosslinkers and catalysts) allow for the flexible control of material properties and mould-/core fabrication, i.e.,: shelf-life: 10–120 min; mould stripping time: 10 min to 24 h; compressive strength: 4–6 MPa (with post-cure: 10–12 MPa); tensile strength: up to 3 MPa (after post-cure). The moulding sands developed achieved thermal resistance temperatures of up to 345 °C, which exceeded that of 280 °C of comparable commercial material. The onset of the thermal decomposition process was 2–3 times longer than that of furan or commercial alkyd/polyester resin. The technology developed allows for the defect-free manufacture of castings (no pinholes) and binder contents minimisation to 1.2–1.5% with quartz and 1.2% with zirconium or chromite sand.

**Motto**: “Skills will remain but a fancy invention; an empty reasoning or idle game unless put to use in the nations’ interest…”. (citate: Dr. S. Staszic; 1755-1826; co-founder of the predecessor of the Polish Academy of Sciences: the Warsaw Royal Society of Friends of Learning)

## 1. Introduction

Nearly 70% of near-net-shape medium and large metallic machinery components are produced by metal castings [1] utilising single-use moulds and cores fabricated from particulate-filled permeable composite materials; moulding sands, in foundry terminology. They comprise 97–99% of compacted refractory filler, e.g., silica, zircon, chromite or olivine sand (typical size: ~150 to 300 μm) bonded by 1–3% of organic or inorganic binder [2].

Moulds and cores are assembled into a three-dimensional (3-D) system of interconnected cavities replicating the net shape of the final casting. Due to their exposure to high temperatures (up to 1500–1600 °C), pressure and erosive action of the heat flux and flow of molten metal, the moulding sand composites must exhibit high-level functional properties such as: (i) adequate strength at ambient and high temperatures to provide structural integrity and dimensional stability of the mould cavity during metal casting and solidification, and (ii) high gas permeability facilitating effective evacuation of gaseous by-products formed within the mould cavity during the decomposition of binder and residual moisture by high-temperature metal.

Meeting the above requirements poses significant scientific and technological challenges, particularly for casting large-size, heavy-weight steel castings weighing up to 30+ tonnes.

## 2. The Structure and Key Properties of Permeable Moulding Sand Composites

The key functional properties of particulate-filled permeable moulding sand composites are controlled by their morphology and chemical composition which, in turn, depend on the following: (i) the filler particles’ chemistry, their size and size distribution and packing density, (ii) type of the binder and inter-particle bonding mechanism(s), e.g.,: physical (van der Waals) interactions or chemical bonding, and (iii) the structure and geometry of binder film or bridges connecting adjacent filler particles.

**Figure 1:**
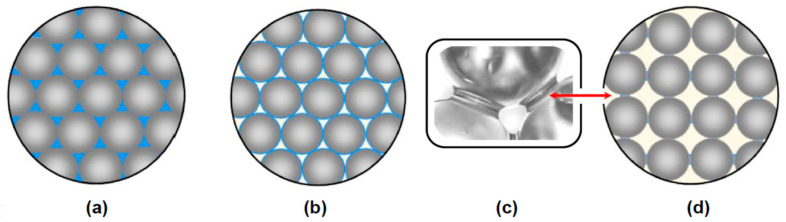
Geometrical aspects of particulate-filled composites morphology controlling their interior 3-D structure and functional properties: engineering composites in comparison with permeable moulding sand composites: (**a**) typical engineering composite comprising: (i) particulate filler compacted to achieve the maximum achievable packing density of reinforcing material (74%) according to face-centred cubic (FCC) packing (or hexagonal closest packing; HCP) manner [3] and (ii) polymeric matrix material (binder: 26%) completely filling all porous cavities between compacted particles; (**b**) FCC-packed permeable composite material (74% filler) with individual filler particles coated by very thin, continuous binder film, as typically observed in moulding sands bonded with liquid organic [1,2,4,5] or inorganic binders [6,7]; (**c**) inter-particle pendular bridges connecting individual filler particles (an example of an FCC-packed permeable composite filled with uniform diameter spherical particles); this is an optimum structure of moulding sand composite facilitating maximisation of composite strength whilst minimising the binder contents; (**d**) inter-particle pendular bridges between filler particles: a permeable composite with cubic lattice compacting, achieving packing density of 52.36% [3].

The geometrical aspects of some of these issues are depicted in Figure 1.

Achieving high-quality, dimensionally accurate castings that exhibit smooth surfaces and are devoid of pinhole defects necessitates the use of suitable binder system enabling: (i) a high level of structural integrity of moulds and cores through adequate strength and thermo-mechanical stability of composite dimensions [8,9] upon exposure to high-temperature and dynamic and static pressures [2] exerted by molten metal in mould cavities, (ii) the fast-speed and low-cost fabrication of moulds and cores, (iii) minimal gaseous by-product formation and kinetics during the binder and residual moisture decomposition [9,10,11,12,13], (iv) adequate composite permeability enabling the rapid evacuation of gaseous by-products evolving in mould cavity upon exposure to liquid metal at the initial stages of its flow and solidification, whilst simultaneously preventing molten metal ingress into inherently porous composite structures, (v) low fracture energy of composites (moulding sand), essential for good mould/core system ‘collapsibility’ (shakeout) during casting removal and cleaning [2] and (vi) the absence of sintered sand residues adhering to the surface of casting.

A broad variety of inorganic and organic (synthetic and bio-based) as well as hybrid binders (organic/inorganic) are used for the fabrication of permeable, particulate-filled composite materials for foundry applications. The analysis of these materials resides outside the scope of this paper and hence, the authors refer readers to comprehensive reviews of this subject available in [2,8,14,15], addressing a broad spectrum of theoretical and practical aspects of currently available and emerging (developmental) liquid binder materials.

## 3. Principal Objective

The principal objective of the research effort presented and analysed in this paper was the development of self-setting, room-temperature-cured alkyd and polyester resins [16,17,18,19,20,21] which were demonstrated to fully satisfy all industrial standards and performance criteria pre-set by the end-user of this technology (Zamech Elbląg/currently ALSTOM Power). This includes all requirements listed in Section 2 above, complemented by the following additional attributes: (i) the effective inhibition of mould/core material shrinkage on exposure to high-temperature metal, (ii) the absence of pinhole defects and (iv) the formation of reducing atmosphere in the mould cavities preventing filler sintering.

**Figure 2:**
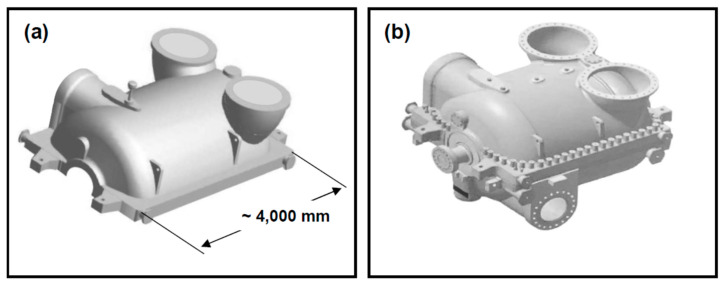
Low-alloy steel castings of turbine casings (Intermediate Pressure (IP) unit of 18K360 condensing steam turbine) manufactured by Zamech under a license from Brown Boveri and Cie (BBC)/currently ALSTOM Power [22,24] with the use of alkyd and polyester resins developed in this work, as installed and operating at Bełchatów Power Generation Plant in Poland [25,26]: (**a**) upper casing, showing approximate dimensions of the casting, and (**b**) the upper and lower casing presented here as integral components of complete assembly of the IP unit of 18K360 turbine (3-D graphics of Figure 1a partially adopted from Ref. [23]).

The developed resins found broad industrial applications as binders for mould- and core-making materials utilised in the production of large-size, complex-shape heavy castings (0.5 to 50 tonnes) made of low-alloy steels [22,23] and cast-iron, such as steam turbine casings (see Figure 2) for power generation plants, naval engine blocks and other heavy-machinery applications.

The research, conceptualized and conducted by the authors of this paper at the Institute of Polymeric Materials Processing and Self-setting Moulding Materials Group of the Technical University of Szczecin/Poland (currently ZUT: West-Pomeranian University of Technology) provided seminal insight into the mechanisms of physico-chemical phenomena controlling mechanical properties and bulk (3-D) microstructure of composites [16,27,28,29,30,31,32]. The practical outcomes of this work were broadly commercialised in the Polish foundry industry [17,18,19,23,24], e.g.,: Ferrum Steel Works/Katowice, Warsaw Steel Works/Warsaw (currently Huta ArcelorMittal), Zabrze Steel Works/Zabrze and Zamech Elblag [22]. The fundamental aspects of this work were further explored with regard to applications in the field of advanced composite systems for automotive, machine and aerospace industries and published [27,28,29,30,31,33] and commercialised through research conducted by both authors at the Institute of Polymers Processing and Recycling at Kassel University/Germany and CSIRO Australia.

To accomplish the objective of this work, the following principal issues were investigated and discussed in this paper: (1) the acceleration of resins cure kinetics for rapid strength development including crosslinking rate control through catalysts addition and, if required, the additional post-cure; (2) the control of the processing time (shelf-life) of composite formulations; (3) thermo-mechanical stability of materials, and (4) kinetics of gaseous by-product emission. The suitability of developed resins was fully verified through extensive pre-production trials under industrial conditions prior to commercial implementation. The technology was implemented by Zamech/ALSTON Power [22,25].

The investigation of fundamental mechanisms controlling the kinetics of binders crosslinking and mechanical and fracture properties of particulate-filled fast-setting composites with furan and other organic and hybrid (organic/inorganic) binder systems provided seminal insight into interfacial phenomena at the solid–liquid interface [27,28,29,30,31,32,33,34] and binder crosslinking [16,17,18,19,20,21]. These, in turn, facilitated the optimisation of composites’ performance, including the minimisation of the binder content (1.2 to 1.5%) through maximising interfacial adhesion [28,33,34,35] and optimising the filler particulate size, rheological and chemical properties of the binder system and composite processing [16,17,35]. The outcomes of this research, which is beyond the scope of this paper, will be a subject of separate publications applicable to emerging transformational technologies such as 3-D inkjet printing of moulds and cores, which currently revolutionize the process of mould and core manufacturing.

## 4. Experimental Section

Alkyd resins and (unsaturated) polyester resins are typically crosslinked by poly-isocyanates (PI). In order to achieve an adequate rate of crosslinking, various catalysts are used: organic compounds of cobalt, zinc, lead and other metals [18,19,20,21] or peroxides [36,37,38,39]. The moulding composite materials with these types of polymeric matrix usually contain up to 1.5% of the binder, of which PIs constitute ~20 to 30%.

The isocyanates (MDI and TDI) with the NCO contents of 30.3 to 48.2% used in this work are denoted as: PI-B; PI-0; PI-1. The catalysts (1.5% solution) investigated in this work were cobalt naphthenate and 1- (β-cyano ethyl)-2-methyl imidazole denoted ‘F’. For comparison, composites (moulding sands) were prepared using commercially available binders: (i) alkyd polyester resin, Georg Fischer [+GF+], denoted as ‘AP’ throughout this paper, crosslinked with an MDI polyisocyanate (Activator 300B, denoted as ‘PI-B’), and (ii) furan resin (Karbafur-Z/40% furfuryl alcohol, denoted as ‘FR’) crosslinked using 40% (in relation to the resin) of phosphoric acid (75%-H_3_PO_4_), or (iii) para-toluene acid (pTSA) used at 30% (in relation to the resin). Table 1 below provides the details of all resins investigated and developed in this work, together with the abovementioned reference materials.

### 4.1. Composites Preparation

i. Equipment and mixing protocol

The amount of binder (polymeric resin + crosslinker) in all formulations was constant at 1.5%. The composite formulations were prepared using an LM-1 laboratory rotary mixer, supplied by the Foundry Institute in Kraków/Poland. The procedure used was as follows: step No. 1—quartz sand and polyisocyanate components were mixed for 2 min, followed by step No. 2—the resin was then added and the entire composition was mixed for a further 5 min.

ii. Composites cure

All composites were cured at 20 °C and were then tested (compressive and/or tensile strength) at the following time intervals lapsing between the completion of composite mixing and sample fabrication: 15′; 20′; 30′; 40′; 1 h; 2 h; 3 h; 5 h and 24 h.

### 4.2. Assessment of Composites Mechanical Properties

The principal mechanical properties of moulding sand composites, such as compressive strength and tensile strength were determined using a designated LRu-2 e mechanical tester supplied by Multiserw-Morek, Wadowice/Poland and tested in accordance with the following standard protocols:

i. *Compressive strength* [40]: determined using cylindrical specimens: Ø50 × 50 mm in accordance with Polish Standard PN 80/H-11073. Foundry Moulding Materials—Measuring of Strength.
ii. *Tensile strength* [41]: determined using dog-bone specimens with square cross-section load-bearing areas of 25.4 × 25.4 mm in accordance with ASTM C307–2018.

The following properties of composites (moulding sands) were investigated:

(a) Cure characteristic

Determined by the strength increase versus time characteristics reflecting the kinetics of resin crosslinking reaction, with an initial onset of the reaction (crosslinking delay) defining the mixture’s shelf life.

(b) Strength
  (i). *Compressive*: using cylindrical specimens: Ø50 × 50 mm [40], and
  (ii). *Tensile*: using dog-bone specimens: load-bearing area = 25.4 × 25.4 mm [41].

These were tested after sample fabrication at the following time intervals lapsing between completion of composite mixing and samples fabrication: 15′; 20′; 30′; 40′; 1 h; 2 h; 3 h; 5 h and 24 h cure at 20 °C.

(c) Shelf-life

The shelf-life was the material processing “window”, i.e., the allowable processing time span lapsing between composite pre-mixing and sample preparation (or core-/mould fabrication) resulting in the cured composite (after 24-h room temperature cure) achieving the strength of no less than 90–95% of those fabricated from a freshly pre-mixed composite material.

(d) Strength after post-cure (1 h at 160 °C)

Determined as follows: (i) hot specimens: tested at 160 °C, and (ii) post-cured (1 h at 160 °C) and then allowed to cool down to 20 °C.

(e) Kinetics of thermal decomposition (gaseous) products evolution

Determined by measuring the rate of gas formation (cm^3^/g vs. time, presented in real time) evolving from composite samples heated from 20 °C to 1000 °C at a rate of 10 °C/min in inert nitrogen atmosphere in an electrically heated tubular muffle furnace (Georg Fisher PGD device).

(f) Thermal resistance
  Determined using the following procedures:
  (i). Determining the highest temperature to which the material can be heated (investigated in the range of 20–400 °C at a heating rate of 10 °C/min) without its strength falling below the initial, i.e., room temperature (20 °C) strength.
    A numerical value of this important parameter was determined from the profile of relationships: compressive strength versus temperature for each system (quartz filler + resin + crosslinking agent) investigated in this paper within the range of 20 °C to 400 °C. In these experiments, cylindrical samples (Ø 50 × 50 mm) were kept for 1 h at the designated elevated temperature, i.e.,: 150, 200, 250, 300, 350 and 400 °C, and were then cooled to 20 °C prior to determining their strength.
  (ii). Using the standard thermogravimetric analysis (TGA).

### 4.3. Filler Properties

The main fractions (80%) of the dried quartz filler (sand) used were in the size range of 0.40/0.32/0.20 mm. The main features of the filler were: (i) average grain size (DIN): d_50_—0.35 mm; (ii) shape factor (non-sphericity): 1.24; (iii) water adsorption: 1.8%; (iv) clay residue contents: 0.6%.

## 5. Mould and Core Materials with Alkyd Resins

**Figure 3:**
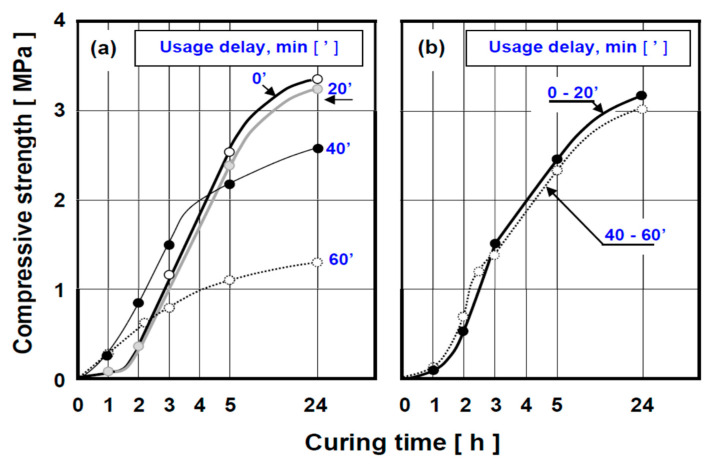
Kinetics of strength increase in composites with alkyd/polyester resin (AP) matrix during a 24-h self-setting period at room temperature (RT = 20 °C). (**a**) presents the properties of commercial formulation [(AP resin) + (PI-B crosslinker)] demonstrating detrimental influence of time delay between mixing completion and sample preparation. The shelf-life of this mixture is 20 min, whilst specimens prepared after 40 or 60 min exhibit final strength reductions by 30% and 60%, respectively after 24-h cure. Data in (**b**) illustrate the properties of the same AP resin crosslinked with our polyisocyanate (PI-0), exhibiting effective shelf-life of 60 min.

Figure 3 below illustrates the kinetics of compressive strength increase in composite samples prepared immediately after mixing and after 20, 40 and 60 min delays using a commercially available alkyd/polyester resin, AP. It is seen from the graphs in this figure that the type of the poly-isocyanate (PI) crosslinker significantly influences the shelf-life and ultimate strength of the fully cured composite material; see detailed comments in the figure caption.

**Figure 4:**
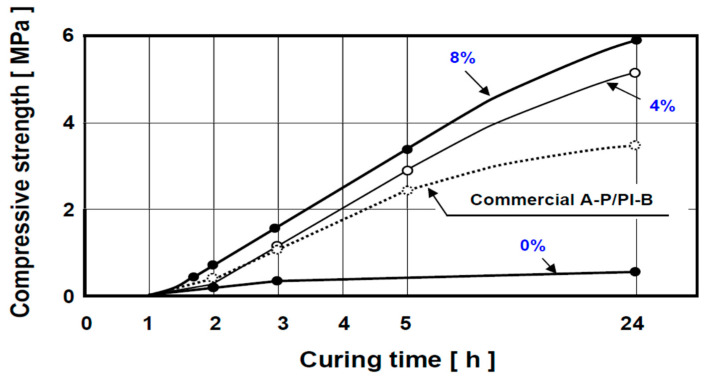
The influence of addition of a catalyst, co-naphthenate (%), to the composite based on alkyd resin A-2K with isocyanate (PI-0) on the kinetics of crosslinking rection and the increase in final strength of the moulding mixture (composite formulation: quartz sand–100 p.w. (parts per weight); A-2K resin–1.14 p.w., and PI-0 isocyanate–0.36 p.w.).

Figure 4, in turn, demonstrates the effectiveness of a catalyst (cobalt naphthanate) in controlling the rate of composite crosslinking when the weight ratio of resin-to-isocyanate is set at the optimum value of 3.1. It is apparent here that the cure of moulding mixture prepared with alkyd resin A-2K and poly-isocyanate PI-0, exhibiting a very slow reaction rate on its own, can be significantly accelerated by this catalyst. It is also seen that increasing the catalyst contents to 4 or 8% significantly accelerates kinetics of crosslinking reaction, whilst simultaneously increasing the ultimate strength of the cured composite.

The shelf-life of the moulding material system illustrated in Figure 4 (A-2K/PI-O) is approximately 60 min. It is also apparent from the graphs that the ultimate strength of our composite is approximately twice as high as that of commercially available reference binder material (AP/PI-B).

## 6. Mould and Core Materials with Polyester Resins

Very favourable strength characteristics and crosslinking kinetics were achieved by all polyester resins developed in this work (UPE 1, UPE 2, UPE 170, UPE 220) cured by the polyisocyanate crosslinker PI-O at the molar ratio NCO/OH = 2. By adding appropriate catalysts, a suitable shelf-life, rapid rate of composite strength increase and its high final strength can also be achieved. Catalyst F proved to be the most effective for this purpose with all resins investigated here.

**Figure 5:**
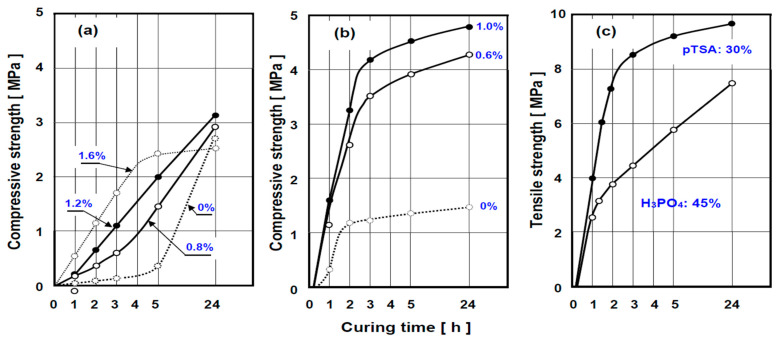
(**a**) By choosing the amount of catalyst F [1- (β-cyano ethyl)-2-methyl imidazole], the processing time (shelf-life) and rate of strength increase in composite with unsaturated polyester resin UPE 2 can be controlled within considerable range; (**b**) rapid cure reaction and high strength can also be achieved with the polyester binder systems: e.g., for UPE1/PI-O with catalyst F; (**c**) cure characteristics of commodity self-setting moulding composite materials utilising furane resin matrix (Karbafur Z) used at 1.5%, cured with either: H_3_PO_4_ or pTSA acid.

As shown in Figure 5a, a relatively slowly curing resin, UPE 2, requires the use of catalysts to meet industrial processing requirements. It can be seen that the crosslinking process is notably accelerated by increasing the amount of a catalyst. An addition of 1.2% of catalyst F (based on the amount of binder) appears to be most advantageous: the composition shelf-life is 20 min, whilst the mould stripping time is approximately 25 to 30 min. If a higher amount of catalyst F is added (1.6%), the strength of material increases rapidly, but the shelf-life shortens to only 10–12 min, whilst the demoulding time is reduced to 5 min.

Amongst all polyester resins developed in our work, UPE 1 resin showed the fastest cure rate and the highest strength, as seen in Figure 5b. When used with the poly-isocyanate, PI-O, the shelf-life of the moulding sand is 12–15 min. The mould stripping time without a catalyst is 20–30 min. The addition of 0.6% of catalyst F reduces it to 10 min, whilst the cured composite strength is increased to about 4 MPa after just 3 h.

For comparative purposes, Figure 5c presents the strength increase (cure) characteristics of commodity self-setting moulding composite materials with furane resin matrix (Karbafur Z, used at 1.5%) cured with H_3_PO_4_ or pTSA acid.

## 7. High-Temperature Strength of Composites Bonded with Self-Setting Alkyd and Polyester Resins

It is well known that the high-temperature strength of self-setting moulding materials made with alkyd resins cured at room temperature is relatively low, so that excessive deformations of large-size cores or mould cavities may occur upon their exposure to molten metal. Detailed chemical analyses demonstrated that the decrease in strength at elevated temperatures for alkyd and polyester binders is mainly caused by allophanate and biuret linkages; their decomposition begins at 110 to 130 °C and is typically completed at 150 °C.

In this paper, we propose that the highest temperature that a heated core-making or moulding material can withstand without its strength falling below that of the initial value (i.e., determined at room temperature) is regarded as the characteristic of its thermal resistance.

**Figure 6:**
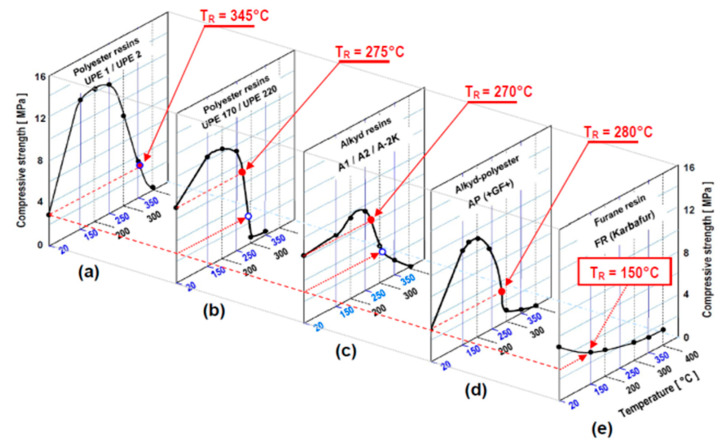
High-temperature strength characteristics of composites with self-setting alkyd and polyester resin matrix (mould-/core-making mixtures) after exposure to elevated temperature in the range of 20 °C to 400 °C (composites comprising: quartz sand + 1.5% of a binder system, as specified by symbols assigned to each individual curve). The specimens were held at designated temperature steps for 1 h and were then tested after cooling to 20 °C. Thermal resistance temperature of each material is marked by the red circle. (*NOTES*: (1) a blue open circle in each resin’s *strength* versus *temperature* characteristic marks the point of thermal resistance temperature of our reference binder system, i.e.,: (AP/PI-B)]; (2) it is noteworthy that for furan resin (FR/Karbafur) a continuous decrease in strength is observed in the entire range of elevated temperatures. Hence, composites (moulding sands) bonded with this particular resin do not exhibit a discrete “thermal resistance” temperature, since any increase of temperature appears to cause their progressive weakening). The characteristics presented here illustrate the strength *versus* temperature profiles of self-setting composites bonded with the following types of polymeric resin matrices: (**a**) polyester resins UPE 1 and UPE 2; (**b**) polyester resins UPE 170 and UPE 220; (**c**) alkyd resins A 1, A 2 and A-2K; (**d**) commercial alkyd-polyester resin A-UPE (+GF+); and (**e**) commercial furane resin FR (Karbafur).

The numerical value of this parameter was determined from individual profiles of relationships: compressive strength versus temperature for each system (quartz filler + resin + crosslinking agent) and was investigated in this paper within the range of 20 °C to 400 °C, as presented in Figure 6. In these experiments, cylindrical samples (Ø 50 × 50 mm) were kept for 1 h at the designated elevated temperature, i.e.,: 150, 200, 250, 300, 350 and 400 °C, and were then cooled to 20 °C prior to determining their compressive strength.

Using the above protocols, the following values were established [16,17] as the characteristic thermal resistance temperatures for all resins investigated and developed in this work:

Polyester resins UPE 1 and UPE 2: 345 °C;

Alkyd/polyester (reference) resin AP: 280 °C;

Polyester resins UPE 170 and UPE 220: 275 °C;

Alkyd resins A 1, A 2, and A-2K: 270 °C.

Similar results were obtained through differential thermal analysis (DTA) tests.

## 8. Effects of Thermal Post-Curing

As seen from experimental data illustrated in Figure 6d, the room temperature (RT = 20 °C) strength of a reference AP alkyd resin (+GF+) investigated in this work is relatively low, which may cause a danger of the excessive deformation of cores or moulds made with this material upon exposure to a molten metal. It is also apparent from high-temperature strength characteristics in Figure 6a–d that all alkyd and polyester binder systems investigated in this work (including the reference system AP) exhibit significant strength increase and retention upon 1 h exposure to temperatures of 150 °C to 250 °C. Considering this observation, we postulated that in industrial applications (especially for large and intricate castings), moulding sands (composites) utilising alkyd and polyester resins should be thermally post-cured.

**Figure 7:**
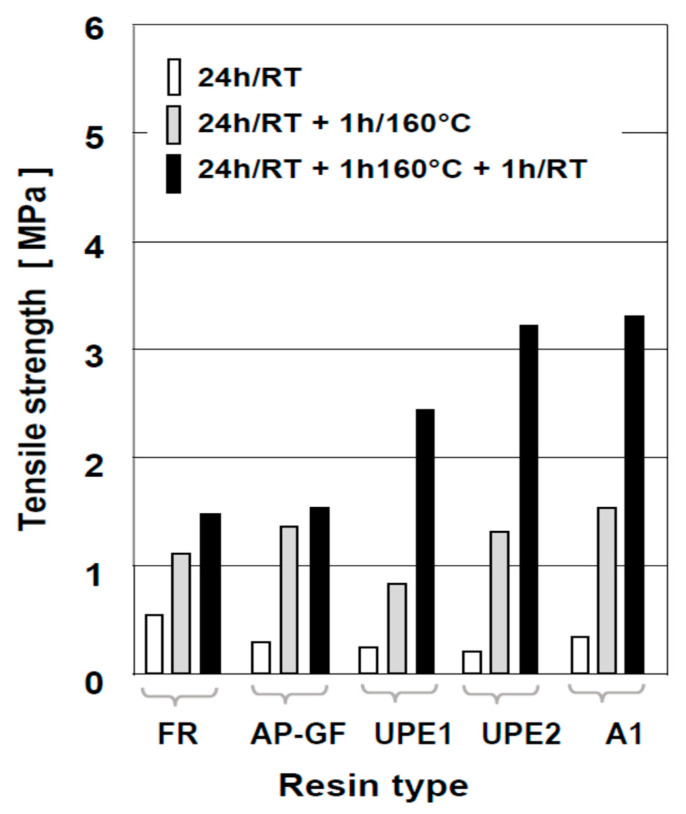
The influence of thermal post-curing on tensile strength of moulding sands made with polyester and alkyd resins investigated in this work. The post-cure and testing conditions are: (i) 24 h→strength after 24 h, after self-setting at room temperature; (ii) 24 h, 160 °C→strength after 24 h self-setting + 1 h post-hardening at 160 °C/RT, tested immediately afterwards; 24 h, 160/20 °C→as in (ii) above, but tested after specimens cooling to room temperature (20 °C) (note: the standard deviation of all results presented in this Figure is in the range of ±5% of the reported mean value).

Figure 7 clearly demonstrates how considerable the strength increase in permeable composites (moulding sands) utilising a polyester or alkyd resin matrix can be if they are post-cured (in the form of test specimens, or actual cores or moulds) for 1 h at 160 °C prior to testing at either elevated or room temperature. Such post-curing not only produces an increase in strength, but also removes considerable volume of volatile components, which reduces the subsequent gas excretion upon mould contact with molten metal. It needs to be noted for the completeness of data regarding properties of our composites that compressive strength of such thermally post-cured materials is approximately 2.5 times higher than tensile strength of each binder systems investigated in this work.

The thermal post-cure of open cores, facilitating the unhindered evacuation of thermal decomposition by-products, is not essential.

## 9. Kinetics of Gas Evolution on Contact with Molten Metal

The point of time when gases begin to evolve from a mould filled with molten metal and the rate at which such gases evolve are of much greater importance in practice than the total amount of gaseous by-products given off by the mould-/core-making mixture.

**Figure 8:**
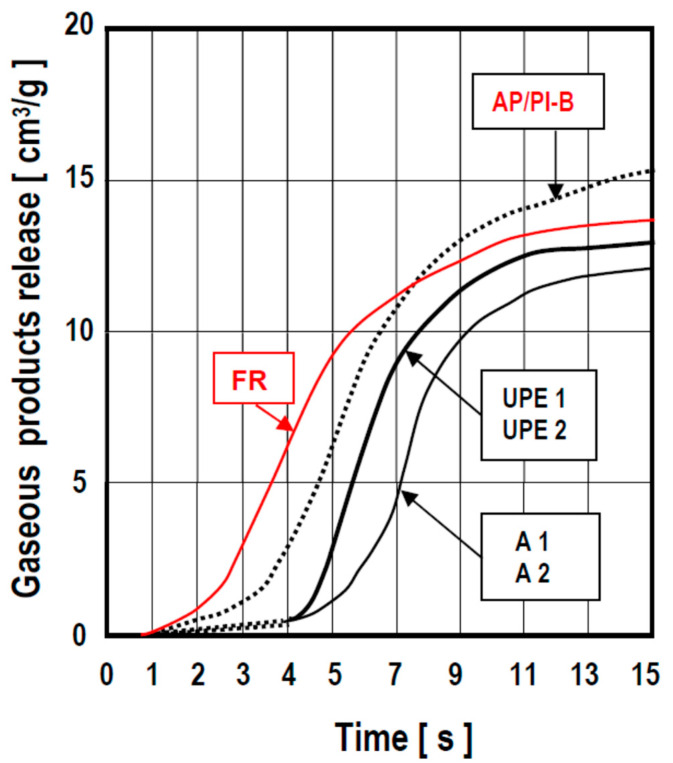
Kinetics of formation of gaseous decomposition by-products from moulding materials utilising a range of binder systems investigated in this work upon contact with molten metal (low-alloy steel).

According to Figure 8, the gas evolution from the decomposition of commercial binders such as furane resin, FR, and alkyd/polyester resin, AP, cured with Activator ‘B’ began to reach a significant level as soon as 2 to 4 s after filling the mould. In contrast to the above results, the evolution of gases from “slow-decomposing” moulding materials bonded by polyester resins PH1, PH2 and alkyd resins AH1, AH2 developed in this work only began to occur when it was essentially over in the case of these much faster decomposing reference materials, i.e., furan (FR) and AP-B binders.

## 10. Industrial Trials

The practical usability of new resins developed through this work was assessed based on extensive tests and observations made at ZAMECH in the course of the production of moulds and cores for low-alloy steel and cast-iron pieces weighing 0.5 to 50 tonnes. The UPE 1, UPE 2 and A 1 resins were used together with the polyisocyanate compound PI-0; if necessary, it was used with the addition of catalyst F. A total of around 20 tonnes of UPE 2 polyester resin and 3 tonnes of both A 1 alkyd resin and UPE 1 polyester resin, were used in these trials. Various types of batch and continuous mixers were used for processing, depending on the operating technology and the size of casting.

It was comprehensively demonstrated that new binders can be used without restriction for small and simple as well as large and intricate castings without rejections occurring. The materials samples taken and tested during production showed very good compliance with the results of earlier laboratory tests.

In the course of all steps of the production process (preparation of the moulding material, mould and core fabrication and casting), the concentration of harmful substances at each station was also determined. It was demonstrated that under appropriate working conditions, the values obtained were always below the permissible limits, and notably, were significantly below these threshold values.

## 11. Conclusions

In this work, it was demonstrated that there is a wide selection of polyester and alkyd resins and crosslinking systems available for use in the fabrication of composite moulds and cores for heavy low-alloy steel castings, depending on the desirable kinetics of strength increase, the composite strength and a material’s processing window (a pre-mixed material’s shelf-life). It has been established that all resins developed in this work are suitable as binders for mould and core materials and they fully comply with the applicable environmental protection regulations. The key observations and conclusions are as follows:

1. Polyester resins UPE 1 and UPE 2 are particularly suitable for use in the preparation of moulding sands designated for heavy-steel castings due to their high strength, good thermal resistance, and very favourable gas evolution kinetics.
2. When using resins denoted as A 1, A 2, UPE 1, UPE 170 and UPE 220, an addition of 1.2% of the binder (resin + polyisocyanate component PI-0) is sufficient, with very good technological properties obtained.
3. By adding catalyst F [1- (β-cyano ethyl)-2-methyl imidazole], the composite crosslinking process (moulding sand hardening) is significantly accelerated without reducing its final strength; at the same time, the composite’s high-temperature strength is also markedly improved. It was observed that the crosslinking rate and ultimate strength of composites with polyester resin UPE 1 and catalyst F [1- (β-cyano ethyl)-2-methyl imidazole] are significantly higher than those observed in any other previously known catalysts.
4. The processing time of the commercially available alkyd polyester resin, AP, can be considerably extended by using the PI-0 polyisocyanate.
5. New binders were also validated as suitable for use with zirconium and chromite sand (applied in monolithic or multi-layered mould/core structures), in which case, the binder content should be around 1.0–1.2% by weight.

## Figures and Tables

**Table 1:** Types and properties of developmental and commercial * resins investigated.

Resin Type	Hydroxyl Value (mg KOH/g)	Acid Value (mg KOH/g)	Iodine Value (g I_2_/100 g)	Viscosity (mPa s)
Polyester, unsaturated (UPE)				
UPE 1	450			1100
UPE 2	205		37	1100
UPE 170	155–180		4	<500
UPE 220	200–230		4	<500
Alkyd (A)				
A 1	35	16	107	<500
A 2	64		105	<500
A-2K	47		101	<500
A-PS	45	18	100	1200
Alkyd/Polyester (A-UPE) *	52	20	77	1830
Furane (F) *				<350

[*] Commercial resins used as benchmark materials.

## Data Availability

The data presented in this study are available on request from the corresponding author.
